# Self-reported and cotinine-verified smoking and increased risk of incident hearing loss

**DOI:** 10.1038/s41598-021-87531-1

**Published:** 2021-04-14

**Authors:** Woncheol Lee, Yoosoo Chang, Hocheol Shin, Seungho Ryu

**Affiliations:** 1 Department of Occupational and Environmental Medicine, Kangbuk Samsung Hospital, Sungkyunkwan University School of Medicine, 29 Saemunan-ro, Jongno-gu, Seoul, 03181 Republic of Korea; 2 Center for Cohort Studies, Total Healthcare Center, Kangbuk Samsung Hospital, Sungkyunkwan University School of Medicine, Seoul, Republic of Korea; 3 Department of Clinical Research Design and Evaluation, SAIHST, Sungkyunkwan University, Seoul, Republic of Korea; 4 Department of Family Medicine, Kangbuk Samsung Hospital, Sungkyunkwan University School of Medicine, Seoul, South Korea

**Keywords:** Biomarkers, Risk factors

## Abstract

We examined the associations of smoking status and urinary cotinine levels, an objective measure of smoking, with the development of new-onset HL. This cohort study was performed in 293,991 Korean adults free of HL who underwent a comprehensive screening examination and were followed for up to 8.8 years. HL was defined as a pure-tone average of thresholds at 0.5, 1.0, and 2.0 kHz ≥ 25 dB in both ears. During a median follow-up of 4.9 years, 2286 participants developed new-onset bilateral HL. Self-reported smoking status was associated with an increased risk of new-onset bilateral HL. Multivariable-adjusted HRs (95% CIs) for incident HL comparing former smokers and current smokers to never-smokers were 1.14 (1.004–1.30) and 1.40 (1.21–1.61), respectively. Number of cigarettes, pack-years, and urinary cotinine levels were consistently associated with incident HL. These associations were similarly observed when introducing changes in smoking status, urinary cotinine, and other confounders during follow-up as time-varying covariates. In this large cohort of young and middle-aged men and women, smoking status based on both self-report and urinary cotinine level were independently associated with an increased incidence of bilateral HL. Our findings indicate smoking is an independent risk factor for HL.

## Introduction

Hearing loss (HL) is an important issue in public health, having rising prevalence and a negative impact on social function and mental and physical health. The World Health Organization (WHO) approximates that around 466 million people had disabling HL in 2015, and over 900 million people will have disabling HL by 2050^1^. Therefore, it is of great importance to develop preventive strategies by identifying modifiable risk factors and individuals at high risk for HL.

Cigarette smoking is a well-known risk factor for a wide range of diseases, but its association with HL has been inconsistent in previous studies. Some studies have suggested a positive association between smoking and HL^2–10^, while others did not^11,12^. However, previous studies have been limited by the ambiguous temporal relationship between smoking and HL due to the cross-sectional design^2,3,5–10^, insufficient sample size^5,6,10–12^, inclusion of mainly male or elderly participants^3,4,6,8^, and lack of adjustment for important confounders (i.e., noise exposure and health behaviors)^3,6^. Furthermore, most studies used subjective measures of smoking, mainly depending on self-report, and the misclassification of smoking status may potentially underestimate the true association of smoking exposure with HL. Since cotinine, a major metabolite of nicotine, is a reliable and objective biomarker that reflects smoking status, it can reduce misclassification bias in self-reporting methods^13,14^. Until now, however, no cohort study has shown the effects of both subjective and objective smoking measures on the development of HL.

Therefore, we investigated the prospective association between smoking status, cigarettes per day, pack-years and risk of developing HL in Korean young and middle-aged adults. In addition, by adding the level of urine cotinine to the smoking parameter, objective reliability could be additionally obtained, while considering time-dependent measures of change in smoking status and other confounders during follow-up.

## Methods

### Study population

**Figure 1:**
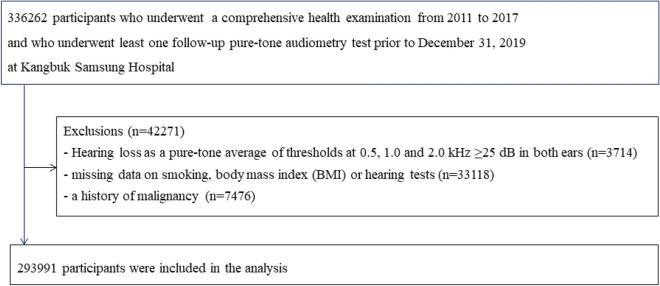
Flow chart of study participants.

This cohort study was done in a subset of the population used in the Kangbuk Samsung Health Study, a cohort study of Korean adults who underwent a comprehensive annual or biennial health examination at the clinics of Kangbuk Samsung Hospital Total Healthcare Screening Center in Seoul and Suwon, South Korea^15^. The current analysis included all study participants who had a comprehensive health screening, including a pure-tone audiometry (PTA) between 2011 and 2017, and at least one follow-up PTA before December 31, 2019 (n = 336,262). After exclusion who met the exclusion criteria (Fig. 1), including HL with an average of PTA thresholds ≥ 25 dB at 0.5, 1.0 and 2.0 kHz in both ears (n = 3714), missing information on smoking, body mass index (BMI), or hearing tests (n = 33,118), or a history of cancer (n = 7,476), a total number of 293,991 participants were analyzed.

We followed the practice of the Declaration of Helsinki and this study was approved by the Institutional Review Board of Kangbuk Samsung Hospital (KBSMC 2020-03-021), which waived the requirement for written informed consent due to the use of anonymized data obtained as part of regular medical examinations.

### Data collection

Physical measurements, hearing tests, and laboratory measurements were performed every 1–2 years. Demographic characteristics, diet, lifestyle factors, and medical history were also collected at each visit using standardized, self-administered questionnaires as previously described^15,16^. Questions regarding smoking, included lifetime and current smoking status, smoking duration, and number of cigarettes per day were included^17^. Participants who had smoked < 100 cigarettes during their lifetime were classified as never-smokers. Participants who had smoked > 100 cigarettes in their lifetime were further categorized as (1) current smokers who smoked currently or (2) former smokers who no longer smoked at the time of their screening examination. Pack-years were categorized as never (0), > 0–10, > 10–20, and ≥ 20 pack-years. Few female participants were identified as having ≥ 20 pack-years and were thus combined with the category of > 10–20 pack-years, resulting in a category of ≥ 10 pack-years in women. Average alcohol consumption was categorized into none, < 20 g of ethanol/day, and ≥ 20 g of ethanol/day^18^. Physical activity level was assessed using the validated Korean version of the International Physical Activity Questionnaire Short Form^19^ and was classified into three categories: inactive, minimally active, and health-enhancing physically active (HEPA)^20^. HEPA status was defined as (i) vigorous intensity activity on three or more days per week accumulating ≥ 1500 metabolic equivalent task (MET) min/week, or (ii) seven days of any combination of walking, moderate intensity, or vigorous intensity activities achieving at least 3000 MET min/week^19^. Cardiovascular disease (CVD) and cancer were defined as physician-diagnosed heart disease or stroke and physician-diagnosed malignancy of any type, respectively^15^.

Height, weight, and blood pressure (BP) were measured by trained nurses. Obesity was defined as BMI ≥ 25 kg/m^2^ according to Asia-specific criteria^21^. Hypertension was defined as a systolic BP ≥ 140 mmHg, a diastolic BP ≥ 90 mmHg, a self-reported history of hypertension, or current use of antihypertensive medications.

Fasting blood measurements included glucose, glycated hemoglobin (HbA1c), lipid profiles, insulin, and hsCRP. Insulin resistance was assessed using the following HOMA-IR equation: fasting blood insulin (uU/mL) × fasting blood glucose (mmol/L)/22.5. Diabetes mellitus (DM) was defined as a fasting serum glucose ≥ 126 mg/dL, HbA1c ≥ 6.5%, a history of physician-diagnosed diabetes, or current use of antidiabetic medications.

Urinary cotinine level was measured using the DRI Cotinine Assay (Microgenics Corp., Fremont, CA, USA) with a modular P800 chemistry analyzer (Roche Diagnostics, Tokyo, Japan). A urine cotinine cut-off point of 100 ng/mL has been used as a reference point in our hospital with analytical measurement range of 34 ng/mL, since nonsmoker urine levels have been reported not to exceed 100 ng/mL in several studies^22,23^. A urinary cotinine level of 50 ng/ml has also been widely used to distinguish tobacco use vs. no tobacco use^24^. Thus, in the present study, urinary cotinine levels were categorized into three groups: (1) < 50 ng/mL, (2) 50 ng/mL to 99 ng/mL, and (3) ≥ 100 ng/mL. Additionally, we performed analysis using categorization of urinary cotinine based on its distribution. Urinary cotinine levels above 50 ng/mL were divided into tertiles (1st tertile, 50–607 ng/mL; 2nd tertile, 608–1303 ng/mL; 3rd tertile, ≥ 1304 ng/mL).

### Audiometric measurements

At baseline and follow-up visits, PTA was performed by trained audiometric technicians using a GSI 67 audiometer (Bedford, MA, USA) equipped with TDH-39 supra-aural earphones (Telephonics Co., Farmingdale, NY, USA) in a dedicated sound-treated booth^25^. Air conduction thresholds were measured in dB hearing level for both ears at 0.5, 1.0, and 2.0 kHz. HL was defined as an average of PTA thresholds ≥ 25 dB at 0.5, 1.0, and 2.0 kHz in bilateral ears.

The Korean Occupational Safety and Health Act 28 requires an annual hearing test at 3.0 and 4.0 kHz in addition to the regular frequencies (0.5, 1.0, and 2.0 kHz) for employees who are exposed to the equivalent sound pressure level of 85 dB(A) over an 8-h work day in the workplace^26^. Hearing tests at 3.0 and 4.0 kHz were performed only in a small proportion of the participants who met the above criteria; thus, these tests were not used to define HL, but as a proxy marker for occupational noise exposure^26^.

### Statistical analysis

The baseline characteristics of the study participants were presented according to smoking status. The primary endpoint was the development of bilateral HL defined as a pure-tone average of thresholds at 0.5, 1.0, and 2.0 kHz ≥ 25 dB in bilateral ears. Each participant was followed from their baseline examination until either the development of bilateral HL or the last health exam conducted prior to December 31, 2019, whichever came first. The incidence rate was calculated as the number of incident cases divided by the number of person-years of follow-up. Since new-onset HL, if it did occur, would have occurred at an unknown time point between the visit at which HL was diagnosed by hearing tests and the prior visit, a parametric proportional hazards model was used to account for this type of interval censoring (stpm command in Stata)^27^.

The hazard ratio (HR) and 95% confidence interval (CI) were calculated for incident HL according to smoking status, cigarettes per day, pack-years smoked, and urinary cotinine levels. Models were initially adjusted for age and sex and then were further adjusted for BMI, alcohol intake (0 g/day, < 20 g/day, ≥ 20 g/day, or unknown), physical activity level (inactive, minimally active, HEPA, or unknown), education level (high school graduate or less, community college or university graduate, graduate school or higher, or unknown), total calorie intake (in quintiles or missing), history of diabetes (no vs. yes), history of hypertension (no vs. yes), history of CVD (no vs. yes), and occupational noise exposure (no vs. yes). To evaluate the effects of changes in smoking status and other covariates over time during follow-up, we conducted additional analyses introducing these variables as time-varying covariates in the models. The proportional hazards assumption was assessed by examining graphs of estimated log (−log) survival; ultimately, no violation of the assumption was found. To test for linear trends, we included the median value of each category (pack-years, cigarettes per day, and urinary cotinine) as a continuous variable in the models.

The sensitivity analyses were performed using different definitions of HL as follows: (1) pure-tone threshold at 0.5 kHz ≥ 25 dB in either ear, (2) pure-tone threshold at 1.0 kHz ≥ 25 dB in either ear, and (3) pure-tone threshold at 2.0 kHz ≥ 25 dB in either ear (separately). Additionally, subgroup analyses were performed by exposure to occupational noise (no vs. yes) and age (< 50 vs. ≥ 50 years).

All analyses were carried out using Stata version 16.0 (Stata Corp LP, College Station, TX, USA). All p-values less than 0.05 were considered to be statistically significant.

## Results

The mean (standard deviation) age of all participants at baseline was 37.8 (8.0) years; 57.4 percent were male; and never, former, and current smokers were reported as 54.4%, 23.7%, and 21.8%, respectively (Table 1). Compared to never-smokers, current smokers were older, more male, and more likely to drink alcohol; to have unhealthy lipid profiles; to have a history of hypertension, diabetes, and CVD; and to have high levels of BMI, BP, glucose, liver enzymes, HOMA-IR, hsCRP, and total energy intake (Table 1). The median urinary cotinine level of current smokers was 818 ng/mL (interquartile range, 282–1445), whereas the median cotinine level of both never and former smokers was 34 ng/mL, which corresponded to the lower limit of the analytical measurement range.

**Table 1:** Baseline characteristics of study participants by smoking status.

Characteristics	Overall	Smoking status			p for trend
		Never smoker	Former smoker	Current smoker	
Number	293,991	160,030	69,822	64,139	
Age (years)^a^	37.8 (8.0)	36.5 (7.8)	40.0 (8.5)	38.9 (7.1)	< 0.001
Male (%)	57.4	29.3	86.7	95.5	< 0.001
Obesity (%)	28.4	19.2	37.1	42.1	< 0.001
Alcohol intake (%)^c^	24.0	10.8	31.8	46.8	< 0.001
HEPA (%)	15.6	14.1	18.9	15.6	< 0.001
High education level (%)^d^	84.4	83.2	86.5	85.3	< 0.001
Hypertension (%)	10.0	6.2	15.2	13.9	< 0.001
Diabetes (%)	3.3	1.9	4.9	5.3	< 0.001
Medication for dyslipidemia (%)	2.0	1.4	3.1	2.3	< 0.001
History of CVD (%)	0.9	0.6	1.4	1.0	< 0.001
Occupational noise (%)	18.6	16.6	25.6	16.1	< 0.001
BMI (kg/m^2^)	23.3 (3.4)	22.4 (3.3)	24.2 (3.1)	24.6 (3.2)	< 0.001
Systolic BP (mmHg)^a^	109.3 (13.0)	105.5 (12.5)	113.7 (12.4)	114.0 (11.8)	< 0.001
Diastolic BP (mmHg)^a^	70.0 (9.9)	67.3 (9.2)	73.2 (9.7)	73.4 (9.6)	< 0.001
Glucose (mg/dL)^a^	94.7 (14.0)	92.3 (11.7)	97.3 (14.9)	98.0 (17.0)	< 0.001
Total cholesterol (mg/dL)^a^	193.3 (34.1)	188.6 (32.7)	198.3 (34.7)	199.7 (34.9)	< 0.001
LDL-C (mg/dL)^a^	120.3 (32.0)	114.7 (30.8)	126.5 (32.2)	127.6 (32.3)	< 0.001
HDL-C (mg/dL)^a^	58.8 (15.4)	62.9 (15.5)	55.6 (14.0)	52.3 (13.4)	< 0.001
Triglycerides (mg/dL)^b^	90 (64–135)	75 (57–106)	105 (74–152)	125 (87–181)	< 0.001
ALT (U/L)^b^	18 (13–28)	15 (11–22)	22 (16–33)	24 (17–36)	< 0.001
GGT (U/L)^b^	20 (13–35)	15 (11–23)	27 (18–44)	34 (22–56)	< 0.001
HOMA-IR^b^	1.22 (0.80–1.81)	1.16 (0.77–1.71)	1.27 (1.84–1.91)	1.31 (0.85–1.98)	< 0.001
hsCRP (mg/L)^b^	0.4 (0.2–0.9)	0.4 (0.2–0.8)	0.5 (0.3–1.0)	0.5 (0.3–1.1)	< 0.001
Total energy intake (kcal/day)^b^^,^^e^	1630.9 (1289.9–2035.4)	1588.3 (1239.0–1999.9)	1640.0 (1307.6–2030.8)	1655.5 (1312.1–2067.6)	< 0.001
Below detection limit of cotinine (%)^f^	78.6	98.7	91.0	11.7	< 0.001
Cotinine level (If > 34)^b^	892 (406–1565)	211 (51–600)	487 (153–1032)	962 (481–1565)	< 0.001
Follow up time (year)^a^	4.8 (2.3)	4.5 (2.2)	5.1 (2.3)	5.1 (2.3)	< 0.001

Data are presented as ^a^means (standard deviation), ^b^median (interquartile range), or percentages.

*ALT* alanine aminotransferase, *BMI* body mass index, *BP* blood pressure, *GGT* gamma glutamyl transferase, *HDL-C* high-density lipoprotein-cholesterol, *HEPA* health-enhancing physically active, *HOMA-IR* homeostasis model assessment of insulin resistance, *hsCRP* high sensitivity C-reactive protein, *LDL-C* low-density lipoprotein-cholesterol.

^c^Alcohol intake ≥ 20 g of ethanol per day.

^d^≥ College graduate.

^e^Among 208,266 subjects with plausible estimated energy intake levels (within three standard deviations from the log-transformed mean energy intake).

^f^Cotinine level ≤ 34 among 207,893 subjects with available cotinine data.

Table 2 shows the relationship between subjective smoking measures and the incidence of bilateral HL. During follow-up of 1,406,455.9 person-years (median 4.9 years; interquartile range, 2.7–6.9 years; maximum 8.8 years), 2286 participants developed new-onset bilateral HL, which corresponds to an incidence rate (95% CI) of 1.6 (1.6–1.7) per 1000 person-years for the overall population, 1.3 (1.2–1.4) per 1000 person-years for women, and 1.8 (1.7–1.9) per 1000 person-years for men. Based on self-report, smoking status was positively associated with an increased risk of developing bilateral HL. Multivariable-adjusted HRs (95% CIs) for incident HL compared to never-smokers for former and current smokers were 1.14 (1.004–1.30) and 1.40 (1.21–1.61), respectively. Increasing baseline smoking-pack-years and smoking intensity, which is measured by the number of cigarettes smoked per day, had a dose–response relationship with the incidence of bilateral HL. For the amount of cigarettes per day, multivariable-adjusted HRs (95% CIs) for incident HL were 1.19 (0.95–1.48), 1.30 (1.13–1.48), and 1.29 (1.11–1.49) compared with < 10, 10–19, and ≥ 20 versus 0 cigarettes per day, respectively (P for trend < 0.001). For pack-years, multivariable-adjusted HRs (95% CIs) for incident HL were 1.00 (0.86–1.16), 1.10 (0.95–1.28), and 1.27 (1.10–1.46) compared with < 10, 10–19.9, and ≥ 20 versus 0 pack-years, respectively (P for trend = 0.001). A similar association was observed between pack-years and incident HL when introducing changes in smoking measures and other confounding factors during follow-up as covariates over time. None of the associations between subjective smoking measures and HL differed by sex (all P for interaction > 0.1), but these associations was not statistically significant among female participants (Supplementary Table S1).

**Table 2:** Development of bilateral hearing loss by subjective measures of smoking status.

Smoking categories	Person-years	Incident cases	Incidence density (per 1000 person-years)	Age/sex adjusted HR (95% CI)	Multivariable-adjusted HR^a^ (95% CI)	HR (95% CI)^b^ in the model using time-dependent variables
**Smoking status**						
Never smoker	723,002.0	906	1.3	1.00 (reference)	1.00 (reference)	1.00 (reference)
Former smoker	358,590.1	782	2.2	1.18 (1.04–1.33)	1.14 (1.004–1.30)	1.17 (1.03–1.34)
Current smoker	324,863.9	598	1.8	1.49 (1.30–1.71)	1.40 (1.21–1.61)	1.43 (1.23–1.65)
p for trend				< 0.001	< 0.001	< 0.001
**Cigarettes per day**						
0	1,081,805.5	1688	1.6	1.00 (reference)	1.00 (reference)	1.00 (reference)
< 10	67,745.2	85	1.3	1.21 (0.97–1.50)	1.19 (0.95–1.48)	0.79 (0.33–1.90)
10–19	171,886.5	286	1.7	1.33 (1.17–1.52)	1.30 (1.13–1.48)	1.35 (0.60–3.02)
≥ 20	83,609.6	225	2.7	1.39 (1.20–1.61)	1.29 (1.11–1.49)	1.60 (0.50–5.09)
p for trend				< 0.001	< 0.001	0.432
**Pack-years**						
0	813,374.7	1083	1.3	1.00 (reference)	1.00 (reference)	1.00 (reference)
< 10	303,025.5	310	1.0	1.02 (0.88–1.18)	1.00 (0.86–1.16)	1.39 (0.94–2.06)
10–19.9	173,005.6	359	2.1	1.17 (1.01–1.35)	1.10 (0.95–1.28)	1.58 (0.75–3.33)
≥ 20	85,285.1	465	5.5	1.39 (1.21–1.59)	1.27 (1.10–1.46)	2.05 (0.75–5.55)
p for trend				< 0.001	0.001	0.016

*BMI* body mass index, *CI* confidence interval, *CVD* cardiovascular disease, *HR* hazard ratio.

^a^Estimated from parametric proportional hazards models. Multivariable model was adjusted for age, sex, center, year of screening exam, BMI, physical activity, alcohol intake, total energy intake, educational level, medication for dyslipidemia, history of CVD, history of diabetes, history of hypertension, and occupational noise exposure.

^b^Estimated from Cox proportional hazards models with smoking category, physical activity, alcohol intake, total energy intake, BMI, medication for dyslipidemia, history of diabetes, history of hypertension, history of cancer, history of CVD, and occupational noise exposure as time-dependent categorical variables and baseline age, sex, center, year of screening exam, and education level as time-fixed variables.

Urinary cotinine levels were associated with an increased risk of developing HL (P for trend < 0.001) (Table 3). Cotinine levels of ≥ 100 ng/ml significantly related with incident HL in the multivariable-adjusted model with a corresponding HR (95% CI) of 1.30 (1.21–1.51). This pattern was observed in both men and women, but there was no significant interaction (P for interaction by sex = 0.891), and the association was not statistically significant among women. In a further time-dependent analysis, the association between cotinine levels and incident HL increased slightly than in the original analyses.

**Table 3:** Development of bilateral hearing loss by urinary cotinine level.

Cotinine level	Person-years (PY)	Incident cases	Incidence density (per 10^3^ PY)	Age sex adjusted HR (95% CI)	Multivariable-adjusted HR^a^ (95% CI)	HR (95% CI)^b^ in the model using time-dependent variables
**Total**						
< 50	803,655.8	670	0.8	1.00 (reference)	1.00 (reference)	1.00 (reference)
50–99	10,655.1	7	0.7	0.76 (0.36–1.61)	0.74 (0.35–1.56)	1.36 (0.72–2.54)
≥ 100	226,151.4	304	1.3	1.40 (1.21–1.62)	1.30 (1.21–1.51)	1.36 (1.16–1.59)
p for trend				< 0.001	< 0.001	< 0.001
**Women**						
< 50	405,889.0	277	0.7	1.00 (reference)	1.00 (reference)	1.00 (reference)
50–99	1662.7	1	0.6	1.18 (0.17–8.38)	1.16 (0.16–8.30)	1.65 (0.23–11.78)
≥ 100	12,889.3	12	0.9	1.49 (0.84–2.66)	1.28 (0.71–2.29)	1.70 (0.97–2.97)
p for trend				0.173	0.401	0.059
**Men**						
< 50	397,766.8	393	1.0	1.00 (reference)	1.00 (reference)	1.00 (reference)
50–99	8992.4	6	0.7	0.72 (0.32–1.61)	0.70 (0.31–1.56)	1.32 (0.68–2.56)
≥ 100	213,262.1	292	1.4	1.39 (1.19–1.62)	1.30 (1.12–1.52)	1.33 (1.13–1.58)
p for trend				< 0.001	0.001	0.001

The p-value for the interaction of sex and cotinine level for risk of hearing loss was 0.891.

*BMI* body mass index, *CI* confidence interval, *CVD* cardiovascular disease, *HR* hazard ratio.

^a^Estimated from parametric proportional hazards models. Multivariable model was adjusted for age, sex (only for total), center, year of screening exam, BMI, physical activity, alcohol intake, total energy intake, educational level, medication for dyslipidemia, history of CVD, history of diabetes, history of hypertension, and occupational noise exposure.

^b^Estimated from Cox proportional hazards models with cotinine level category, physical activity, alcohol intake, total energy intake, BMI, medication for dyslipidemia, history of diabetes, history of hypertension, history of cancer, history of CVD, and occupational noise exposure as time-dependent categorical variables and baseline age, sex, center, year of screening exam, and education level as time-fixed variables.

In the analysis using categories based on tertiles of urinary cotinine, multivariable-adjusted HRs (95% CI) for incident HL comparing 1st, 2nd and 3rd tertiles to low urinary cotinine of < 50 ng/ml were 1.10 (0.87–1.39), 1.35 (1.09–1.67) and 1.37 (1.12–1.69), respectively (Table 4). This pattern was similarly observed in both men and women. In the analysis using urinary cotinine as a continuous variable, multivariable-adjusted HR (95% CI) for incident HL was 1.013 (1.001–1.025) per 100 unit increase in cotinine level in overall subjects.

**Table 4:** Development of bilateral hearing loss by categories of urinary cotinine based on its distribution.

Cotinine level	Person-years (PY)	Incident cases	Incidence density (per 10^3^ PY)	Age sex adjusted HR (95% CI)	Multivariable-adjusted HR^a^ (95% CI)	HR (95% CI)^b^ in the model using time-dependent variables
**Total**						
< 50	803,655.8	670	0.8	1.00 (reference)	1.00 (reference)	1.00 (reference)
1st tertile (50–607)	81,178.1	82	1.0	1.16 (0.92–1.47)	1.10 (0.87–1.39)	1.40 (1.10–1.77)
2nd tertile (608–1303)	79,061.4	109	1.4	1.45 (1.17–1.78)	1.35 (1.09–1.67)	1.32 (1.04–1.67)
3rd tertile (≥ 1304)	76,567.1	120	1.6	1.49 (1.21–1.82)	1.37 (1.12–1.69)	1.36 (1.08–1.71)
p for trend				< 0.001	< 0.001	0.001
Per 100 unit increase in cotinine level ^c^				1.011 (0.999–1.023)	1.013 (1.001–1.025)	1.010 (1.003–1.017)
**Women**						
< 50	405,889.0	277	0.7	1.00 (reference)	1.00 (reference)	1.00 (reference)
1st tertile (50–607)	7,885.9	4	0.5	0.94 (0.35–2.51)	1.17 (0.92–1.50)	1.14 (0.42–3.06)
2nd tertile (608–1303)	4,222.8	5	1.2	1.93 (0.80–4.66)	1.42 (1.15–1.77)	2.14 (0.88–5.19)
3rd tertile (≥ 1304)	2,443.4	4	1.6	1.97 (0.73–5.29)	1.47 (1.19–1.81)	2.07 (0.84–5.08)
p for trend				0.077	0.271	0.029
Per 100 unit increase in cotinine level ^c^				1.003 (0.927–1.085)	1.003 (0.923–1.090)	1.042 (0.973–1.117)
**Men**						
< 50	397,766.8	393	1.0	1.00 (reference)	1.00 (reference)	1.00 (reference)
1st tertile (50–607)	73,292.2	78	1.1	0.87 (0.32–2.33)	1.11 (0.87–1.42)	1.40 (1.10–1.80)
2nd tertile (608–1303)	74,838.6	104	1.4	1.81 (0.74–4.38)	1.34 (1.07–1.66)	1.28 (1.00–1.63)
3rd tertile (≥ 1304)	74,123.7	116	1.6	1.40 (0.51–3.85)	1.37 (1.11–1.69)	1.32 (1.04–1.68)
p for trend				< 0.001	0.001	0.004
Per 100 unit increase in cotinine level ^c^				1.011 (0.999–1.023)	1.012 (0.001–1.025)	1.008 (0.997–1.019)

The p-value for the interaction of sex and cotinine level for risk of hearing loss was 0.880.

*BMI* body mass index, *CI* confidence interval, *CVD* cardiovascular disease, *HR*, hazard ratio,* CI* confidence interval, *HR* hazard ratio.

^a^Estimated from parametric proportional hazards models. Multivariable model was adjusted for age, sex (only for total), center, year of screening exam, BMI, physical activity, alcohol intake, total energy intake, educational level, medication for dyslipidemia, history of CVD, history of diabetes, history of hypertension, and occupational noise exposure.

^b^Estimated from Cox proportional hazards models with cotinine level category, physical activity, alcohol intake, total energy intake, BMI, medication for dyslipidemia, history of diabetes, history of hypertension, history of cancer, history of CVD, and occupational noise exposure as time-dependent categorical variables and baseline age, sex, center, year of screening exam, and education level as time-fixed variables.

^c^Among current smokers.

In a stratified subgroup analysis by occupational noise exposure (Supplementary Table S2), the association of smoking status, cigarettes per day, pack-years, and cotinine level with the risk of incident HL was consistently observed in participants without exposure to occupational noise. The average age of the HL group was 51.1 (standard deviation, 11.2) years. In the subgroup analysis by age (< 50 vs. ≥ 50 years), the association between self-reported smoking and incident HL was stronger in younger individuals aged < 50 years than in older individuals (p for interaction < 0.05) (Supplementary Table S2).

Sensitivity analyses were performed using different definitions of HL as follows: (1) pure-tone threshold at 0.5 kHz ≥ 25 dB in either ear, (2) pure-tone threshold at 1.0 kHz ≥ 25 dB in either ear^28^ and (3) pure-tone threshold at 2.0 kHz ≥ 25 dB in either ear (separately) (Supplementary Tables S3-S4). The incidence rate (95% CI) of HL at 0.5 kHz, 1.0 kHz, and 2.0 kHz were 4.9 (4.8–5.0) per 1000 person-years, 3.2 (3.1–3.3) per 1000 person-years, and 12.5 (12.3–12.8), respectively. In these sensitivity analyses, the associations of smoking status, cigarettes per day, pack-years, and cotinine level with incident HL were similar to in the original analyses, which HL defined as a pure-tone average of thresholds at 0.5, 1.0, and 2.0 kHz ≥ 25 dB in bilateral ears.

## Discussion

In this longitudinal study of young and middle-aged adults in Korea, current smoking, cigarettes per day, and pack-years were significantly associated with an increased risk of developing HL. In addition, a significant association was found between HL and urine cotinine levels, which are objective smoking biomarker. The significance of the above associations were maintained even after adjusting for various confounders, including occupational noise exposure.

Previous longitudinal studies were limited by short follow-up periods^4,12,29^, small sample sizes^6,11,12,29^, audiometric measurements of single individual low frequency or self-reported hearing^4,30,31^, and inclusion of mainly male or elderly participants^4,6,29,30^. A previous meta-analysis including 4 cohort studies reported that the risk ratio (95% CI) for HL in current smokers was 1.97 (1.44, 2.70) and in former smokers was 1.49 (0.93, 2.39)^32^. In a recent cohort study of 1925 participants with a 15-year follow up, current smokers were more likely to cause HL than never-smokers (HR = 1.31, 95% CI = 1.003–1.71 in a model adjusted for age, sex, and education), but detailed information on smoking intensity, smoking duration, and other confounders was not available in this study^33^. In another cohort study of over 50,000 workers aged 20–64 years in Japan, the self-reported smoking status (current, former), smoking intensity (cigarettes smoked per day), and smoking pack-years were all significantly associated with an elevated risk of HL at an individual frequency (defined as > 30 dB at 1 kHz and > 40 dB at 4 kHz) after multivariable adjustment^28^. However, this study population was mainly composed of male workers (~ 85%), and information regarding important confounders including occupational noise exposure, alcohol drinking, and physical activity was only available in a subset of the study population^28^. In our study, we tried to reduce the measurement error by using the traditional standard definition of HL as an average of PTA thresholds ≥ 25 dB at 0.5, 1.0, and 2.0 kHz in bilateral ears^34^, rather than at one individual frequency in either ear. In addition, ≥ 25 dB was used as the cut-off threshold for HL, as opposed to the > 30 dB value used in the previous study^28^, enabling us to capture mild HL. Moreover, on the assumption that smoking causes HL through a systemic pathway^35,36^, we defined bilateral HL as the primary endpoint instead of unilateral HL, which may also be caused by a localized infection or accident, and found that current smoking status, intensity, and pack-years were still significantly associated with bilateral HL. When we repeated the analysis using the same definition as in the previous study for comparison^28^, the associations between smoking parameters and incident HL were consistently observed. We adopted urine cotinine as another measure of smoking to reduce the misclassification bias in the self-report method. and reaffirmed the robustness of our study findings on the associations between smoking and incident HL. Furthermore, all these associations were consistently observed in a time-dependent analysis that took into account changes over time in smoking parameters and other confoundings.

We found an association between smoking and low-frequency HL, defined as an average threshold of 0.5, 1.0, and 2.0 kHz. Previous studies have shown that smoking is associated with HL at both low and high frequencies^9,37^. Some studies have reported that the association of smoking with HL was stronger at high frequencies than at low frequencies^8,35^. In contrast, a Danish study reported that cardiovascular risk factors, including smoking, are related to low-frequency HL but not to high-frequency HL^38^. Studies using objective measures of auditory function, such as otoacoustic emission, also supported the link between smoking and hearing at various frequencies^39,40^. Smoking can cause negative alterations in the cochlea, leading to different effects on the base and apex of the cochlea via microvascular compromise and induced hypoxemia^35,36^. These pieces of evidence may describe that smoking can be associated with HL in the low-frequency range.

The associations between smoking and incidence of HL did not statistically differ by sex in our study, but the association was not statistically significant among women. The women’s smoking prevalence was very low, 7.4% being former smokers and 2.3% being current smokers, compared to the prevalence among men being 35.9% for former smokers and 36.3% for current smokers. Thus, the inconclusive association for women may be explained by the small number of smokers, which is insufficient to provide precise estimates. On the other hand, epidemiological studies have also reported that smoking-associated HL was more pronounced in men than in women^31,41^. A study of 85 healthy volunteers aged 25–45 years using otoacoustic emissions (OAEs), a test for activity in the outer hair cells of the cochlea, demonstrated a significant decline in OAEs among male smokers but not among the females, suggesting that men are more susceptible to smoking-induced adverse change in the organ of hearing^39^. These sex differences might be explained by the potential protective effect of female sex hormones in hearing, as previously proposed^39,42^. Future research is needed to interpret the mechanism between smoking and HL, while considering possible differences by sex.

The average age of the HL group was higher than the group without incident HL but the association between self-reported smoking and incident HL was stronger in younger individuals aged < 50 years than in older individuals. The reason for this difference is unclear. Participants aged ≥ 50 years may have more comorbidities than younger counterparts and relative contribution of smoking to incident HL might be lower in older age group. Due to the use of multiple comparisons, another explanation for the observed difference across age group can be attributed simply due to chance.

The strength of this study is that it is a large cohort, allows longitudinal analysis, and includes standardized laboratory data of a comprehensive health examination at all visits. And, unlike previous studies, urine cotinine provides less biased results compared to the self-reported smoking status. Our subjects were relatively healthy and young (average age, 37.8 years old), with a low occupational noise exposure rate (18.6%). Therefore, the findings are less likely to be biased by age-related HL and occupational noise exposure than previous studies conducted in older populations or those exposed to occupational noise.

There are some limitations to this study. First, hearing results at high frequencies above 2.0 kHz were not available. However, since HL was defined in the spectrum below 2.0 kHz, which is mainly used by human voices in real life, the association between smoking and hearing could be investigated with only low-frequency data. Second, leisure exposure, which is the main cause of noise-induced HL, was not measured at a comprehensive examination. However, since noise-induced HL is mainly related to high-frequency HL above 3.0 kHz, there was no significant interference even without leisure exposure data in our study. Third, information on ototoxic drugs (i.e., cisplatin and aminoglycoside antibiotics) was not available. However, by excluding subjects with a history of cancer, we tried to minimize the effects of ototoxic substances such as anticancer drugs. Fourth, even though we used cotinine level as an objective measure of smoking status, the biological half-life of cotinine is only 19 to 40 h in the body^14^. Therefore, measurement errors cannot be avoided in irregular smokers and persons who intentionally did not smoke temporarily^17^. Fifth, detailed analyses on secondhand smoking were not possible in our study even though secondhand smoking has been reported to be associated with HL and can affect urine cotinine levels^43^. While previous studies have suggested a cut-off for distinguishing between active and secondhand smokers of between 20 and 100 ng/mL^23,44^, the lower limit of detection of urinary cotinine was 34 ng/mL in our study. This limited our ability to further examine the dose–response relationship of urinary cotinine with incident HL at low levels of urinary cotinine that could correspond to secondhand smoking. Therefore, the effect of unmeasured secondhand smoke cannot be excluded. Additionally, we cannot ignore the possibility of bias related to residual confounding in regard to measured or unmeasured confounders in the associations observed in the present study. Lastly, our results were derived from young and healthy Korean adults, thus limiting the generalizability of our findings on different populations with various age and race/ethnicity.

In conclusion, smoking status in both subjective self-report and objective urinary cotinine levels were independently associated with increased risk of HL in a dose–response manner, after adjustment for confounders including occupational noise exposure. This association was evident even in individuals without occupational noise exposure. Our findings support the conclusion that smoking is an independent cause for HL even in young adults, underscoring the importance of smoking control on HL, in addition to the wide range of other diseases attributable to smoking.

## Supplementary Information

Supplementary information.

## Abbreviations

ALT: Alanine aminotransferase
AST: Aspartate aminotransferase
BMI: Body mass index
CI: Confidence interval
HOMA-IR: Homeostasis model assessment of insulin resistance
HR: Hazard ratio
HDL-C: High-density lipoprotein cholesterol
hsCRP: High sensitivity C-reactive protein
LDL-C: Low-density lipoprotein cholesterol
