# Supplementary material for: Self-reported and cotinine-verified smoking and increased risk of incident hearing loss
Source: Sci Rep. 2021 Apr 14;11:8103. doi: 10.1038/s41598-021-87531-1 (PMC8047000; doi:10.1038/s41598-021-87531-1)
Supplement: Supplementary file 1 — Supplementary information. [file 41598_2021_87531_MOESM1_ESM.docx]

**Self-reported and cotinine-verified smoking and increased risk of incident hearing loss**

Woncheol Lee^1^, Yoosoo Chang^1,2,3^**^*^**, Hocheol Shin^2,4^, Seungho Ryu^1,2,3^**^*^**

^1^Department of Occupational and Environmental Medicine, Kangbuk Samsung Hospital, Sungkyunkwan University School of Medicine, Seoul, Republic of Korea

^2^Center for Cohort Studies, Total Healthcare Center, Kangbuk Samsung Hospital, Sungkyunkwan University School of Medicine, Seoul, Republic of Korea

^3^Department of Clinical Research Design & Evaluation, SAIHST, Sungkyunkwan University, Seoul, Republic of Korea

^4^Department of Family Medicine, Kangbuk Samsung Hospital, Sungkyunkwan University School of Medicine, Seoul, South Korea

**^*^Corresponding authors:**

**Seungho Ryu, MD, PhD,**

Department of Occupational and Environmental Medicine, Kangbuk Samsung Hospital

Sungkyunkwan University School of Medicine

Samsung Main Building B2, 250, Taepyung-ro 2ga, Jung-gu, Seoul, Korea 04514

Telephone: +82-2-2001-5137, Fax: +82-2-757-0436

Email address: sh703.yoo@gmail.com

**^*^Co-corresponding author**:

**Yoosoo Chang, MD, PhD**

Department of Occupational and Environmental Medicine, Kangbuk Samsung Hospital

Sungkyunkwan University School of Medicine

Samsung Main Building B2, 250, Taepyung-ro 2ga, Jung-gu, Seoul, Korea 04514

Telephone: +82-2-2001-5139, Fax: +82-2-757-0436

Email address: yoosoo.chang@gmail.com

Supplementary Table S1. Hazard ratios (95% CIs) for bilateral hearing loss by sex, smoking status, number of cigarettes per day, and pack-years

|  | Person-years (PY) | Number of events | Incidence rate  (1000 PY) | Age-adjusted HR (95% CI) | Multivariable-adjusted HR^a^  (95% CI) | HR (95% CI) ^b^ in model using time-dependent variables |
| --- | --- | --- | --- | --- | --- | --- |
| **Women (n = 125388)** | | | | | | |
| Smoking status |  |  |  |  |  |  |
| Never smoker | 513809.3 | 675 | 1.3 | 1.00 (reference) | 1.00 (reference) | 1.00 (reference) |
| Former smoker | 49041.6 | 74 | 1.5 | 1.30 (1.03–1.66) | 1.29 (1.01–1.64) | 1.42 (1.12–1.80) |
| Current smoker | 12941.8 | 21 | 1.6 | 1.48 (0.96–2.28) | 1.37 (0.89–2.12) | 1.16 (0.70–1.95) |
| *p for trend* |  |  |  | 0.007 | 0.018 | 0.017 |
| Cigarettes smoked / day |  |  |  |  |  |  |
| 0 | 562865.3 | 749 | 1.3 | 1.00 (reference) | 1.00 (reference) | 1.00 (reference) |
| <10 | 8329.1. | 9 | 1.1 | 1.26 (0.65–2.43) | 1.15 (0.60–2.23) | 0.79 (0.33–1.90) |
| ≥10 | 4299.2 | 12 | 2.8 | 1.75 (0.99–3.09) | 1.62 (0.91–2.88) | 1.42 (0.73–2.77) |
| *p for trend* |  |  |  | 0.044 | 0.100 | 0.486 |
| Pack-years |  |  |  |  |  |  |
| 0 | 535842.5 | 720 | 1.3 | 1.00 (reference) | 1.00 (reference) | 1.00 (reference) |
| <10 | 28950.0 | 32 | 1.1 | 1.49 (1.04–2.13) | 1.41 (0.99–2.03) | 1.39 (0.94–2.06) |
| ≥10 | 2284.6 | 10 | 4.4 | 1.62 (0.89–3.03) | 1.50 (0.80–2.82) | 1.72 (0.94–3.14) |
| *p for trend* |  |  |  | 0.010 | 0.031 | 0.017 |
| **Men (n =168603)** | | | | | | |
| Smoking status |  |  |  |  |  |  |
| Never smoker | 209192.6 | 231 | 1.1 | 1.00 (reference) | 1.00 (reference) | 1.00 (reference) |
| Former smoker | 309548.5 | 708 | 2.3 | 1.13 (0.97–1.31) | 1.09 (0.94–1.27) | 1.11 (0.96–1.29) |
| Current smoker | 311922.1 | 577 | 1.8 | 1.45 (1.24–1.69) | 1.36 (1.16–1.58) | 1.38 (1.18–1.62) |
| *p for trend* |  |  |  | <0.001 | <0.001 | < 0.001 |
| Cigarettes smoked / day |  |  |  |  |  |  |
| 0 | 518940.2 | 939 | 1.8 | 1.00 (reference) | 1.00 (reference) | 1.00 (reference) |
| <10 | 59416.1 | 76 | 1.3 | 1.19 (0.94–1.50) | 1.20 (0.95–1.52) | 1.20 (0.95–1.52) |
| 10–19 | 168187.8 | 276 | 1.6 | 1.31 (1.14–1.50) | 1.28 (1.12–1.47) | 1.26 (1.09–1.45) |
| ≥20 | 83009.1 | 223 | 2.7 | 1.38 (1.19–1.60) | 1.27 (1.09–1.47) | 1.33 (1.13–1.56) |
| *p for trend* |  |  |  | <0.001 | <0.001 | <0.001 |
| Pack-years |  |  |  |  |  |  |
| 0 | 277532.2 | 363 | 1.3 | 1.00 (reference) | 1.00 (reference) | 1.00 (reference) |
| <10 | 274075.5 | 278 | 1.0 | 0.94 (0.81–1.10) | 0.95 (0.81–1.11) | 0.93 (0.80–1.10) |
| 10–19.9 | 171018.0 | 353 | 2.1 | 1.13 (0.98–1.31) | 1.07 (0.92–1.24) | 1.10 (0.95–1.27) |
| ≥20 | 84988.0 | 461 | 5.4 | 1.37 (1.19–1.57) | 1.22 (1.05–1.41) | 1.22 (1.06–1.41) |
| *p for trend* |  |  |  | <0.001 | 0.003 | 0.002 |

The p-value for the interaction of sex and smoking status for bilateral hearing loss was 0.528 (multivariable-adjusted model).

The p-value for the interaction of sex and cigarettes smoked/day for bilateral hearing loss was 0.775 (multivariable-adjusted model).

The p-value for the interaction of sex and pack-years for bilateral hearing loss was 0.165 (multivariable-adjusted model).

^a^ Estimated from Cox proportional hazards models using age as a time scale to estimate HRs and 95% CIs. Multivariable model was adjusted for age (timescale), center, year of screening exam, smoking status, regular exercise, BMI, education level, history of diabetes, history of hypertension, and history of cardiovascular disease.

^b^ Estimated from Cox proportional hazards models with smoking status, alcohol consumption, regular exercise, history of diabetes, history of hypertension, history of cardiovascular disease, and BMI as time-dependent categorical variables and baseline age, sex, center, year of screening exam, and education level as time-fixed variables.

Abbreviations: BMI, body mass index; CI, confidence interval; HR, hazard ratio.

Supplementary Table S2. Hazard ratios ^a^ (95% CIs) for bilateral hearing loss by smoking status, number of cigarettes per day, pack-years and cotinine level categories according to occupational noise exposure and age

| Smoking, pack-years, and cotinine level categories | Exposure to occupational noise | | p for interaction | Age | | p for interaction |
| --- | --- | --- | --- | --- | --- | --- |
|  | No exposure to occupational noise  (n=239246) | Exposure to occupational noise (n=54745) |  | <50 years  (n=270,372) | ≥50 years  (n=23,619) |  |
| Smoking status |  |  | 0.12 |  |  | <0.001 |
| Never smoker | 1.00 (reference) | 1.00 (reference) |  | 1.00 (reference) | 1.00 (reference) |  |
| Former smoker | 1.17 (1.02–1.34) | 1.00 (0.78–1.28) |  | 1.48 (1.26-1.74) | 1.06 (0.90-1.24) |  |
| Current smoker | 1.46 (1.26–1.69) | 1.08 (0.81–1.44) |  | 1.72 (1.46-2.03) | 1.07 (0.88-1.29) |  |
| *p for trend* | <0.001 | 0.565 |  | <0.001 | 0.598 |  |
| Cigarettes per day |  |  | 0.211 |  |  | <0.001 |
| 0 | 1.00 (reference) | 1.00 (reference) |  | 1.00 (reference) | 1.00 (reference) |  |
| <10 | 1.31 (1.03–1.66) | 0.73 (0.40–1.33) |  | 1.04 (0.80-1.37) | 1.10 (0.75-1.60) |  |
| 10–19 | 1.32 (1.14–1.53) | 1.17 (0.86–1.60) |  | 1.37 (1.16-1.61) | 1.01 (0.81-1.27) |  |
| ≥20 | 1.33 (1.14–1.56) | 1.03 (0.69–1.54) |  | 1.71 (1.41-2.08) | 0.98 (0.79-1.22) |  |
| *p for trend* | <0.001 | 0.591 |  | <0.001 | 0.967 |  |
| Pack-years |  |  | 0.14 |  |  | <0.001 |
| 0 | 1.00 (reference) | 1.00 (reference) |  | 1.00 (reference) | 1.00 (reference) |  |
| <10 | 1.07 (0.92–1.26) | 0.71 (0.51–0.98) |  | 0.95 (0.79-1.13) | 0.96 (0.76-1.20) |  |
| 10–19.9 | 1.12 (0.96–1.31) | 1.03 (0.76–1.39) |  | 1.70 (1.42-2.03) | 0.92 (0.75-1.13) |  |
| ≥20 | 1.29 (1.12–1.50) | 1.15 (0.85–1.55) |  | 2.77 (2.27-3.37) | 1.16 (0.98-1.37) |  |
| *p for trend* | <0.001 | 0.326 |  | <0.001 | 0.027 |  |
| Cotinine level |  |  | 0.19 |  |  | 0.594 |
| <50 | 1.00 (reference) | 1.00 (reference) |  | 1.00 (reference) | 1.00 (reference) |  |
| 50–99 | 0.95 (0.45–2.00) | - |  | 0.73 (0.33-1.64) | 0.77 (0.11-5.48) |  |
| ≥100 | 1.39 (1.18–1.63) | 0.99 (0.70–1.39) |  | 1.34 (1.14-1.58) | 1.12 (0.81-1.54) |  |
| *p for trend* | <0.001 | 0.884 |  | 0.001 | 0.494 |  |

^a^ Estimated from parametric proportional hazards models. Multivariable model was adjusted for age, sex, center, year of screening exam, BMI, physical activity, alcohol intake, total energy intake, educational level, medication for dyslipidemia, history of CVD, history of diabetes, and history of hypertension.

During 1120694 years of follow-up, 1928 individuals without exposure to occupational noise developed HL (incidence rate of 1.7 per 1000 person-years). During 285761.9 years of follow-up, 358 cases of HL developed among those with (incidence rate of 1.3 per 1000 person-years).

During 1318078.0 years of follow-up, 1156 individuals aged <50 years developed HL (incidence rate of 0.9 per 1000 person-years), while during 88377.9 years of follow-up, 1130 cases of HL developed among older subjects (incidence rate of 12.8 per 1000 person-years).

Supplementary Table S3. Development of hearing loss ^a^ by smoking status, number of cigarettes per day, pack-years, and cotinine level.

| **Category** | **Multivariable-adjusted HR^b^ (95% CI)** | | | | | |
| --- | --- | --- | --- | --- | --- | --- |
|  | **Hearing loss (1kHz)** | | | **Hearing loss (2kHz)** | | |
| **Total** | **Total (n=279743)** | **Women** | **Men** | **Total (n=279743)** | **Women** | **Men** |
| Smoking status |  |  |  |  |  |  |
| Never smoker | 1.00 (reference) | 1.00 (reference) | 1.00 (reference) | 1.00 (reference) | 1.00 (reference) | 1.00 (reference) |
| Former smoker | 1.16 (1.06–1.27) | 0.97 (0.81–1.17) | 1.26 (1.13–1.41) | 1.10 (1.03–1.17) | 0.96 (0.83–1.10) | 1.15 (1.07–1.24) |
| Current smoker | 1.27 (1.15–1.40) | 1.11 (0.80–1.55) | 1.35 (1.20–1.51) | 1.20 (1.12–1.29) | 1.11 (0.86–1.43) | 1.24 (1.15–1.34) |
| *p for trend* | < 0.001 | 0.805 | < 0.001 | < 0.001 | 0.886 | < 0.001 |
| Cigarettes per day |  |  |  |  |  |  |
| 0 | 1.00 (reference) | 1.00 (reference) | 1.00 (reference) | 1.00 (reference) | 1.00 (reference) | 1.00 (reference) |
| < 10 | 1.05 (0.90–1.22) | 1.05 (0.67–1.65) | 1.04 (0.88–1.22) | 1.01 (0.91–1.13) | 1.21 (0.87–1.67) | 0.99 (0.88–1.11) |
| 10–19 | 1.14 (1.03–1.25) | 1.15 (0.65–2.04) | 1.12 (1.02–1.24) | 1.12 (1.05–1.20) | 0.97 (0.61–1.54) | 1.11 (1.04–1.19) |
| ≥ 20 | 1.20 (1.08–1.35) | 1.37 (0.44–4.28) | 1.20 (1.07–1.34) | 1.19 (1.10–1.29) | 1.25 (0.52–3.01) | 1.19 (1.10–1.29) |
| *p for trend* | < 0.001 | 0.473 | 0.001 | < 0.001 | 0.506 | <0.001 |
| Pack-years |  |  |  |  |  |  |
| 0 | 1.00 (reference) | 1.00 (reference) | 1.00 (reference) | 1.00 (reference) | 1.00 (reference) | 1.00 (reference) |
| < 10 | 1.08 (0.98–1.19) | 0.81 (0.60–1.08) | 1.12 (1.003–1.24) | 1.01 (0.94–1.09) | 0.91 (0.74–1.12) | 1.02 (0.95–1.11) |
| 10–19.9 **^c^** | 1.13 (1.02–1.26) | 0.96 (0.50–1.85) | 1.18 (1.05–1.31) | 1.21 (1.12–1.30) | 0.94 (0.57–1.54) | 1.22 (1.13–1.31) |
| ≥ 20 **^c^** | 1.20 (1.07–1.35) |  | 1.28 (1.13–1.45) | 1.18 (1.09–1.30) |  | 1.20 (1.10–1.30) |
| *p for trend* | 0.001 | 0.235 | < 0.001 | <0.001 | 0.416 | < 0.001 |
| Cotinine level |  |  |  |  |  |  |
| < 50 | 1.00 (reference) | 1.00 (reference) | 1.00 (reference) | 1.00 (reference) | 1.00 (reference) | 1.00 (reference) |
| 50–99 | 0.95 (0.64–1.41) | 1.01 (0.31–3.13) | 0.94 (0.62–1.44) | 0.82 (0.60–1.12) | 1.27 (0.57–2.84) | 0.77 (0.55–1.08) |
| ≥ 100 | 1.14 (1.03–1.25) | 1.28 (0.91–1.82) | 1.13 (1.02–1.24) | 1.17 (1.09–1.25) | 1.08 (0.80–1.46) | 1.17 (1.09–1.26) |
| *p for trend* | 0.009 | 0.166 | 0.019 | <0.001 | 0.548 | <0.001 |

^a^ Hearing loss was defined as ≥25dB in either ear at 1000 and 2000 Hz.

^b^ Estimated from parametric proportional hazards models. Multivariable model was adjusted for age, sex (only for total), center, year of screening exam, BMI, physical activity, alcohol intake, total energy intake, educational level, medication for dyslipidemia, history of CVD, history of diabetes, history of hypertension, and occupational noise exposure.

^c^ ≥10 pack-years for women

Supplementary table S4. Development of hearing loss ^a^ by smoking status, number of cigarettes per day, pack-years and cotinine levels

| **Category** | **Multivariable-adjusted HR^b^ (95% CI)** | | |
| --- | --- | --- | --- |
|  | **Hearing loss (0.5kHz)** | | |
| **Total** | **Total** | **Women** | **Men** |
| Smoking status |  |  |  |
| Never smoker | 1.00 (reference) | 1.00 (reference) | 1.00 (reference) |
| Former smoker | 0.99 (0.92-1.07) | 0.82 (0.70-0.97) | 1.05 (0.96-1.14) |
| Current smoker | 1.16 (1.07-1.26) | 1.24 (0.96-1.60) | 1.20 (1.10-1.31) |
| *p for trend* | < 0.001 | 0.673 | < 0.001 |
| Cigarettes per day |  |  |  |
| 0 | 1.00 (reference) | 1.00 (reference) | 1.00 (reference) |
| < 10 | 1.08 (0.96-1.23) | 1.22 (0.86-1.73) | 1.07 (0.93-1.22) |
| 10–19 | 1.13 (1.04-1.22) | 1.06 (0.66-1.71) | 1.13 (1.04-1.22) |
| ≥ 20 | 1.27 (1.17-1.39) | 1.71 (0.81-3.60) | 1.27 (1.16-1.39) |
| *p for trend* | < 0.001 | 0.154 | < 0.001 |
| Pack-years |  |  |  |
| 0 | 1.00 (reference) | 1.00 (reference) | 1.00 (reference) |
| < 10 | 0.97 (0.89-1.05) | 0.97 (0.77-1.22) | 0.96 (0.88-1.05) |
| 10–19.9 **^c^** | 1.05 (0.97-1.14) | 1.23 (0.79-1.92) | 1.07 (0.98-1.16) |
| ≥ 20 **^c^** | 1.15 (1.05-1.26) |  | 1.20 (1.10-1.32) |
| *p for trend* | 0.001 | 0.667 | < 0.001 |
| Cotinine level |  |  |  |
| < 50 | 1.00 (reference) | 1.00 (reference) | 1.00 (reference) |
| 50–99 | 0.99 (0.71-1.39) | 1.03 (0.39-2.76) | 0.99 (0.69-1.42) |
| ≥ 100 | 1.21 (1.12-1.31) | 0.98 (0.70-1.37) | 1.23 (1.13-1.33) |
| *p for trend* | < 0.001 | 0.927 | < 0.001 |

^a^ Hearing loss was defined as ≥25dB in either ear at 500 Hz

^b^ Estimated from parametric proportional hazard models. The multivariable model was adjusted for age, sex (only for total), center, year of screening exam, BMI, physical activity, alcohol intake, total energy intake, educational level, medication for dyslipidemia, history of CVD, history of diabetes, history of hypertension, and occupational noise

^c^ ≥10 pack-years for women

Abbreviations: CI, confidence interval; HR, hazard ratio

**Supplementary Table S5. Development of hearing loss and explanatory factors**

|  | Multivariate-adjusted HR (95% CI) |
| --- | --- |
|  |  |
| Age per 5-year increment | 1.95 (1.91-1.99) |
| Sex |  |
| Women | 1.00 (reference) |
| Men | 0.92 (0.80-1.05) |
| Smoking status |  |
| Never smoker | 1.00 (reference) |
| Former smoker | 1.14 (1.00–1.30) |
| Current smoker | 1.40 (1.21–1.61) |
| Alcohol intake |  |
| 0 g/day | 1.00 (reference) |
| <20 g/day | 1.13 (0.99–1.29) |
| ≥20 g/day | 1.27 (1.09–1.49) |
| Physical activity |  |
| Inactive | 1.00 (reference) |
| Minimal | 1.02 (0.93–1.13) |
| HEPA | 1.02 (0.91–1.14) |
| Education level |  |
| < College graduate | 1.00 (reference) |
| ≥ College graduate | 0.75 (0.67–0.83) |
| BMI per 1 kg/m^2^ increment | 1.04 (1.02~1.05) |
| Total energy intake quintiles |  |
| Q1 | 1.00 (reference) |
| Q2 | 0.89 (0.71–1.11) |
| Q3 | 0.87 (0.70–1.08) |
| Q4 | 0.80 (0.65–0.99) |
| Q5 | 0.82 (0.67–1.00) |
| History of CVD |  |
| No | 1.00 (reference) |
| Yes | 0.92 (0.80-1.05) |
| History of hypertension |  |
| No | 1.00 (reference) |
| Yes | 1.03 (0.92-1.55) |
| History of diabetes |  |
| No | 1.00 (reference) |
| Yes | 1.40 (1.22-1.61) |
| Medication for hyperlipidemia |  |
| No | 1.00 (reference) |
| Yes | 0.99 (0.85-1.17) |
| Occupational noise |  |
| No | 1.00 (reference) |
| Yes | 0.97 (0.86-1.08) |

NOTE. Multivariate models are adjusted for all other variables listed for the model.
